# Tridimensional Regression for Comparing and Mapping 3D Anatomical Structures

**DOI:** 10.1155/2012/604543

**Published:** 2011-10-04

**Authors:** Kendra K. Schmid, David B. Marx, Ashok Samal

**Affiliations:** ^1^Department of Biostatistics, College of Public Health, University of Nebraska Medical Center, 984375 Nebraska Medical Center, Omaha, NE 68198-4375, USA; ^2^Department of Statistics, University of Nebraska-Lincoln, 340 Hardin Hall North, Lincoln, NE, 68583-0963, USA; ^3^Department of Computer Science and Engineering, University of Nebraska-Lincoln, Lincoln, NE 68588-0115, USA

## Abstract

Shape analysis is useful for a wide variety of disciplines and has many applications. There are many approaches to shape analysis, one of which focuses on the analysis of shapes that are represented by the coordinates of predefined landmarks on the object. This paper discusses Tridimensional Regression, a technique that can be used for mapping images and shapes that are represented by sets of three-dimensional landmark coordinates, for comparing and mapping 3D anatomical structures. The degree of similarity between shapes can be quantified using the tridimensional coefficient of determination (*R* ^2^). An experiment was conducted to evaluate the effectiveness of this technique to correctly match the image of a face with another image of the same face. These results were compared to the *R* ^2^ values obtained when only two dimensions are used and show that using three dimensions increases the ability to correctly match and discriminate between faces.

## 1. Introduction

Tobler [1] proposed bidimensional regression as a tool for computing the degree of similarity between two planar configurations of points and to estimate mapping relations between two objects that are represented by a set of two-dimensional landmarks. Bidimensional regression is an extension of linear regression where both dependent and independent variables are represented by coordinate pairs, instead of scalar values. Specifically, Tobler [1] suggested that bidimensional regression may be useful for comparing signatures, geographical maps, or faces. The latter was done in the context of face recognition by Shi et al. [2] and Kare et al. [3]. 

Tobler's [1] method has been extended to *Tridimensional Regression* for situations when both dependent and independent variables are represented by three-dimensional coordinates [4]. The purpose of this paper is to provide a summary of that extension, to illustrate the use of tridimensional regression for comparing and mapping anatomical structures, and to compare the effectiveness of the two-dimensional and three-dimensional methods. Widespread use of three-dimensional imaging devices in many areas of research makes this research timely. This technique is broadly applicable to any situation where spatial configurations of three-dimensional points are compared. Specific instances where tridimensional regression may be of use are three-dimensional mapping and comparison of objects or structures that are represented by their three-dimensional coordinates. The *R* ^2^ values derived from regression allow the degree of similarity between two objects to be quantified.

## 2. Methods

In this section, a brief summary of bidimensional regression and its extension to three dimensions is provided. Details of the tridimensional regression models are provided.

### 2.1. Bidimensional Regression

Nakaya [5] defines a bidimensional regression model as

(1) $$\left(\begin{array}{c} u_{i}\\ v_{i} \end{array}\right)=\left(\begin{array}{c} g\left(x_{i},y_{i}\right)\\ h\left(x_{i},y_{i}\right) \end{array}\right)+\left(\begin{array}{c} \varepsilon_{i}\\ \eta_{i} \end{array}\right),$$

where (*u*_*i*_, *v*_*i*_) is the dependent variable, (*x*_*i*_, *y*_*i*_) represent the corresponding coordinates of the independent variable, *g* and *h* are transformation functions used to estimate mapping relations between independent and dependent variables, and (*ε*_*i*_, *η*_*i*_) is an error vector that is assumed to be normally and independently distributed. Both Tobler [1] and Nakaya [5] discuss obtaining estimates for parameters in *g* and *h* using the method of least-squares so that

(2) $$\overset{n}{\underset{i=1}{\sum }}\left[{\left(u_{i}-\hat{g}\left(x_{i},y_{i}\right)\right)}^{2}+{\left(v_{i}-\hat{h}\left(x_{i},y_{i}\right)\right)}^{2}\right],$$

where $\hat{g}$ and $\hat{h}$ are the transformation functions evaluated at the parameter estimates, is minimized. Here *n* is the number of landmark points used in the analysis. The normal equations are obtained in the usual manner [1], and by solving ${\hat{\beta }}_{j}$ in

(3) $$X_{j}^{T}X_{j}{\hat{\beta }}_{j}=X_{j}^{T}Y,$$

where **X**_*j*_ is the design matrix of transformation *j* and $Y={\left[\begin{array}{cc} u & v \end{array}\right]}^{T}$ is a (2*n* × 1) vector for the dependent variable partitioned by the coordinates, will yield the least-squares parameter estimates [6]. The design matrix (**X**_*j*_) will depend on the transformation used and the number of parameters to estimate; hence, the dimension of ${\hat{\beta }}_{j}$ will also be determined by the type of transformation.

Tobler [1] proposes four bidimensional regression models, three of which are intrinsically linear and one is curvilinear. Friedman and Kohler [7] argue that the curvilinear model may be too general for practical use and describe the linear transformations in more detail. Each of the other three transformations is linearized by reparameterization prior to solving the parameter estimates. 

The three linear transformations yield the Euclidean, affine, and projective models where in each model the original coordinates are scaled, rotated, and translated. These transformations form a hierarchy with the Euclidean being the simplest (fewest parameters) and the projective the most complex (most parameters) of the models. 

**Figure 1:**
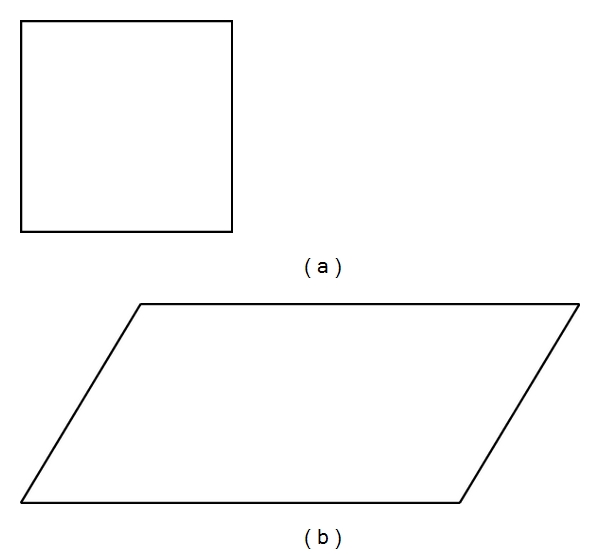
Example of a bidimensional affine transformation.

**Figure 2:**
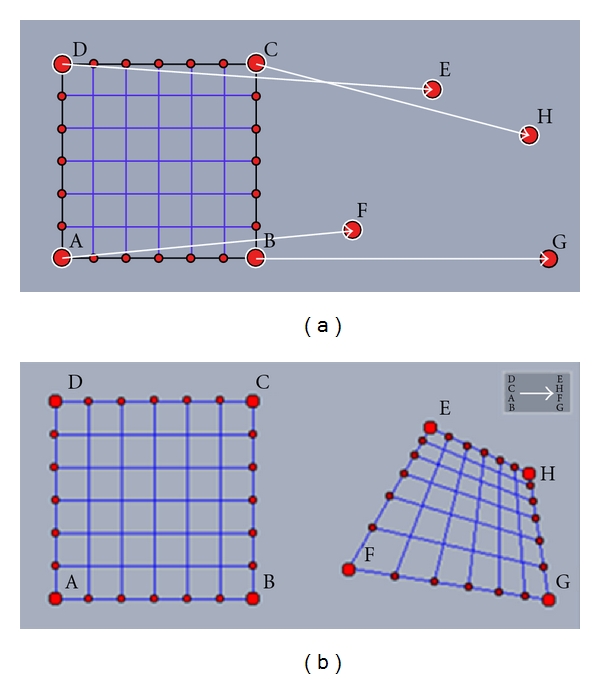
Example of a bidimensional projective transformation (ABCD→FGHE) [9].

Details of bidimensional regression models can be found in [1, 5, 7, 8]. Briefly, the Euclidean model is a similarity transformation in that the overall shape remains unchanged. The coordinates are translated, rotated, and isotropically scaled [8], thus preserving the original shape and angles. The affine model allows for *X* and *Y* coordinates to be scaled independently, and the configuration could exhibit shear (*γ*) (e.g., a square may become a parallelogram; Figure 1). The projective transformation, which is the most complex, allows the size, shape, and orientation to change as a function of viewpoint [7]. An example of a projective transformation is shown in Figure 2. 

In the Euclidean and affine transformations, the models are linearized by reparameterization, and then the normal equations can be derived in the usual manner. Once the parameters have been estimated [7], provide equations for calculating the scale and rotation values for the Euclidean transformation and the scale, shear, and rotation values for the affine transformation. 

The equations for the projective transformation can be rewritten using homogeneous coordinates and put in matrix notation as shown in (16). Homogeneous coordinates can be used with any of the models to provide a uniform framework for all transformations. For rotation, scaling, and shear, the transformed coordinates can be expressed as the product of a transformation matrix and the original coordinates. For translation, however, the coordinates are derived by addition of the translation vector to the original coordinates. Use of homogeneous coordinates makes all the transformations multiplicative. This is accomplished by adding an additional coordinate (*t*), called the homogeneous coordinate. 

The homogeneous coordinate is added purely for mathematical simplification and has no effect on the transformation of coordinates. For example, it is convenient to represent a sequence of transformations as the product of the corresponding transformation matrices. Thus, in the Euclidean and affine models, the translation parameters become multiplicative and one matrix could be used for all of the transformation parameters [10]. With the projective model, the conversion is used to linearize the model, and once the object is mapped using homogeneous coordinates, the original coordinates are restored by dividing by the homogeneous coordinate, *t*. However, when this is done, the restriction placed on *t* results in parameter estimates of ${\hat{\beta }}_{31}=0$, ${\hat{\beta }}_{32}=0,\;\;\text{and}\;{\hat{\beta }}_{33}=1$. Consequently, the projective transformation is reduced to the affine transformation and the results are identical. The conversion to homogeneous coordinates is adequate for determining the location of transformed points, but not for obtaining transformation parameter estimates. If left in terms of the original equations, the parameters of the projective transformation can be estimated using nonlinear regression. When extended to three dimensions, a similar approach is used.

The similarity of the two objects is assessed using the bidimensional correlation coefficient [1],

(4) $$R_{2D}=\sqrt{1-\frac{\sum_{i}\left\{{\left(u_{i}-{\hat{u}}_{i}\right)}^{2}+{\left(v_{i}-{\hat{v}}_{i}\right)}^{2}\right\}}{\sum_{i}\left\{{\left(u_{i}-\overset{̅}{u}\right)}^{2}+{\left(v_{i}-\overset{̅}{v}\right)}^{2}\right\}}}.$$

### 2.2. Tridimensional Regression

The bidimensional regression models proposed by Tobler [1] can be extended to instances where three-dimensional data are used for comparison. A specific instance may include anatomical structures that are represented by three-dimensional landmark coordinates, but tridimensional regression can be useful for determining the degree of similarity between any two objects that are represented by three-dimensional coordinates.

In this paper, the linear transformations discussed by Tobler [1] will be extended to three dimensions. Extensions to the Euclidean, affine, and projective transformations are described in detail where the dependent and independent variables are represented by their three-dimensional coordinates,

(5) $$\left(\begin{array}{c} u_{i}\\ v_{i}\\ w_{i} \end{array}\right),\;\;\left(\begin{array}{c} x_{i}\\ y_{i}\\ z_{i} \end{array}\right),\;\text{respectively}.$$

#### 2.2.1. Euclidean Transformation

The three-dimensional Euclidean transformation is similar to the two-dimensional case in that coordinates are simply translated, rotated, and isotropically scaled. The overall shape and the angles of the original object are preserved, and parallel lines in the original object are mapped to parallel lines in the transformed space. There is an additional translation parameter, and the rotation matrix differs depending on which axis(es) are used for the rotation. In general, the number of rotation parameters is *k*(*k* − 1)/2, where *k* is the number of dimensions. Therefore, there are three rotation parameters for the general three-dimensional Euclidean transformation. However, for instances when it is known that all three rotations are not necessary, the transformation can be reduced to one or two rotations. These special cases are discussed in detail in [4].

**Three-Dimensional Euclidean Transformation with One Angle of Rotation**

The format of the rotation matrix depends on the axis of rotation. The formats for each of the three rotations are shown below, where *γ* is the angle of rotation about the *x*-axis, *θ* is the angle of rotation about the *y*-axis, and *ϕ* is the angle of rotation about the *z*-axis:

(6) $$\begin{array}{c} R_{X}=\left[\begin{array}{ccc} 1 & 0 & 0\\ 0 & \cos\gamma & -\sin\gamma \\ 0 & \sin\gamma & \cos\gamma \end{array}\right],\\ R_{Y}=\left[\begin{array}{ccc} \cos\theta & 0 & \sin\theta \\ 0 & 1 & 0\\ -\sin\theta & 0 & \cos\theta \end{array}\right],\\ R_{Z}=\left[\begin{array}{ccc} \cos\phi & -\sin\phi & 0\\ \sin\phi & \cos\phi & 0\\ 0 & 0 & 1 \end{array}\right]. \end{array}$$

  The general form of the three-dimensional Euclidean transformation is

(7) $$\left[\begin{array}{c} u_{i}\\ v_{i}\\ w_{i} \end{array}\right]=\left[\begin{array}{c} \alpha_{1}\\ \alpha_{2}\\ \alpha_{3} \end{array}\right]+sR\left[\begin{array}{c} x_{i}\\ y_{i}\\ z_{i} \end{array}\right],$$

where **R** is one of the rotation matrices.

As in the two-dimensional case, the transformation can be linearized by reparameterization, where the new transformation matrix (**R**′) is a combination of the scale and rotation parameters. The reparameterized transformations and their normal equations follow.

For rotation about the *x-*axis,

(8) $$\begin{array}{c} R_{X}^{\prime }=\left[\begin{array}{ccc} 1 & 0 & 0\\ 0 & \beta_{1} & -\beta_{2}\\ 0 & \beta_{2} & \beta_{1} \end{array}\right],\\ u_{i}=\alpha_{1}+x_{i},\\ v_{i}=\alpha_{2}+\beta_{1}y_{i}-\beta_{2}z_{i},\\ w_{i}=\alpha_{3}+\beta_{2}y_{i}+\beta_{1}z_{i}, \end{array}$$

and deriving the normal equations in the usual manner yields

(9) $$\begin{array}{cc} & \left[\begin{array}{ccccc} \text{N} & 0 & 0 & 0 & 0\\ 0 & \text{N} & 0 & \sum y_{i} & -\sum z_{i}\\ 0 & 0 & \text{N} & \sum z_{i} & \sum y_{i}\\ 0 & \sum y_{i} & \sum z_{i} & \sum \left(y_{i}^{2}+z_{i}^{2}\right) & 0\\ 0 & -\sum z_{i} & \sum y_{i} & 0 & \sum \left(y_{i}^{2}+z_{i}^{2}\right) \end{array}\right]\left[\begin{array}{c} \alpha_{1}\\ \alpha_{2}\\ \alpha_{3}\\ \beta_{1}\\ \beta_{2} \end{array}\right]\\ & \;\;=\left[\begin{array}{c} \sum \left(u_{i}-x_{i}\right)\\ \sum v_{i}\\ \sum w_{i}\\ \sum \left(v_{i}y_{i}+w_{i}z_{i}\right)\\ \sum \left(w_{i}y_{i}-v_{i}z_{i}\right) \end{array}\right]. \end{array}$$

Similar details for rotation about the *y* and *z* axes can be found in [4].

**Three-Dimensional Euclidean Transformation with Multiple Angles of Rotation**

When more than one rotation is used, the reparameterization to linearize the model is not obvious; therefore, the rotation matrices remain in terms of the rotation parameters and nonlinear regression is used. The advantage of using nonlinear regression is that the rotation and scale parameters are directly estimated instead of being solved in terms of *β*_*i*_; the disadvantage in using nonlinear regression is convergence may not be reached and starting values must be specified. The similarity of the two objects is assessed using the Pseudo*- R* ^2^ as defined by [11]. The Pseudo-*R* ^2^ is calculated in the same manner as *R* ^2^, but, in general, is not guaranteed to be greater than zero. Again, the rotation matrix differs depending upon the axes of rotation. An example of a two-rotation Euclidean transformation is shown below.

For rotation about *x* and *y* axes,

(10) $$\left[\begin{array}{c} u_{i}\\ v_{i}\\ w_{i} \end{array}\right]=\left[\begin{array}{c} \alpha_{1}\\ \alpha_{2}\\ \alpha_{3} \end{array}\right]+s\left[\begin{array}{ccc} \cos\theta & 0 & \sin\theta \\ \sin\gamma \sin\theta & \cos\gamma & -\sin\gamma \cos\theta \\ -\cos\gamma \sin\theta & \sin\gamma & \cos\gamma \cos\theta \end{array}\right]\left[\begin{array}{c} x_{i}\\ y_{i}\\ z_{i} \end{array}\right].$$

 In the general form of the three-dimensional Euclidean transformation, the order in which the transformations are applied will result in different parameter estimates. Permuting this order will result in different estimates of the rotation parameters, but the measure of similarity will remain the same regardless of the order of transformations. The following system of equations shows the rotations in the order of *x*-axis, *y*-axis, and then *z*-axis:

(11) $$\begin{array}{cc} \left[\begin{array}{c} u_{i}\\ v_{i}\\ w_{i} \end{array}\right] & =\left[\begin{array}{c} \alpha_{1}\\ \alpha_{2}\\ \alpha_{3} \end{array}\right]+s\left[\begin{array}{ccc} \cos\theta \cos\phi & -\cos\theta \sin\phi & \sin\theta \\ \sin\gamma \sin\theta \cos\phi +\cos\gamma \sin\phi & -\sin\gamma \sin\theta \sin\phi +\cos\gamma \cos\phi & -\sin\gamma \cos\theta \\ -\cos\gamma \sin\theta \cos\phi +\sin\gamma \sin\phi & \cos\gamma \sin\theta \sin\phi +\sin\gamma \cos\phi & \cos\gamma \cos\theta \end{array}\right]\left[\begin{array}{c} x_{i}\\ y_{i}\\ z_{i} \end{array}\right]. \end{array}$$

#### 2.2.2. Affine Transformation

**Figure 3:**
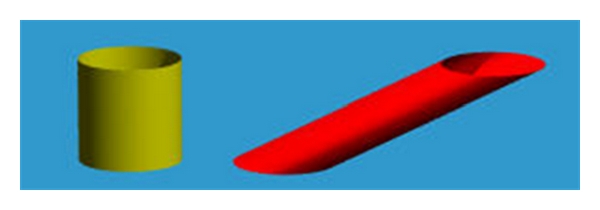
Example of a tridimensional affine transformation [12].

The extension of the affine transformation from two dimensions into three dimensions includes additional parameters for translation, scaling, rotation, and shear.  Figure 3 shows an example of a three-dimensional affine transformation. The transformed coordinates in affine transformations are given by

(12) $$\begin{array}{c} u_{i}=\alpha_{1}+\beta_{11}x_{i}+\beta_{12}y_{i}+\beta_{13}z_{i},\\ v_{i}=\alpha_{2}+\beta_{21}x_{i}+\beta_{22}y_{i}+\beta_{23}z_{i},\\ w_{i}=\alpha_{3}+\beta_{31}x_{i}+\beta_{32}y_{i}+\beta_{33}z_{i},\\ \left[\begin{array}{c} u_{i}\\ v_{i}\\ w_{i} \end{array}\right]=\left[\begin{array}{c} \alpha_{1}\\ \alpha_{2}\\ \alpha_{3} \end{array}\right]+\left[\begin{array}{ccc} \beta_{11} & \beta_{12} & \beta_{13}\\ \beta_{21} & \beta_{22} & \beta_{23}\\ \beta_{31} & \beta_{32} & \beta_{33} \end{array}\right]\left[\begin{array}{c} x_{i}\\ y_{i}\\ z_{i} \end{array}\right]. \end{array}$$

Deriving the normal equations in the usual manner yields

(13) $$\begin{array}{cc} & \;\begin{array}{c} \left[\begin{array}{cc} I_{3}⊗\text{N} & I_{3}⊗\left(\begin{array}{ccc} \sum x_{i} & \sum y_{i} & \sum z_{i} \end{array}\right)\\ I_{3}⊗\left(\begin{array}{c} \sum x_{i}\\ \sum y_{i}\\ \sum z_{i} \end{array}\right) & I_{3}⊗\left(\begin{array}{ccc} \sum x_{i}^{2} & \sum x_{i}y_{i} & \sum x_{i}z_{i}\\ \sum x_{i}y_{i} & \sum y_{i}^{2} & \sum y_{i}z_{i}\\ \sum x_{i}z_{i} & \sum y_{i}z_{i} & \sum z_{i}^{2} \end{array}\right) \end{array}\right]\left[\begin{array}{c} \alpha_{1}\\ \alpha_{2}\\ \alpha_{3}\\ \beta_{11}\\ \beta_{12}\\ \beta_{13}\\ \beta_{21}\\ \beta_{22}\\ \beta_{23}\\ \beta_{31}\\ \beta_{32}\\ \beta_{33} \end{array}\right] \end{array}\\ & \;\;=\left[\begin{array}{c} \sum u_{i}\\ \sum v_{i}\\ \sum w_{i}\\ \sum u_{i}x_{i}\\ \sum u_{i}y_{i}\\ \sum u_{i}z_{i}\\ \sum v_{i}x_{i}\\ \sum v_{i}y_{i}\\ \sum v_{i}z_{i}\\ \sum w_{i}x_{i}\\ \sum w_{i}y_{i}\\ \sum w_{i}z_{i} \end{array}\right], \end{array}$$

where **I**_3_ is a 3 × 3 identity matrix and ⊗ is the direct product of the two matrices. 

#### 2.2.3. Projective Transformation

The extension of the projective transformation from two to three dimensions involves the conversion to homogeneous coordinates (16). Additional parameters are added corresponding to the coordinate of the third dimension. In a projective transformation, the size, shape, and orientation can all change as a function of viewpoint. While this is a nonlinear transformation, by using homogeneous coordinates, the model can be linearized in order to obtain the normal equations and estimate the parameters. The equations to obtain the transformed coordinates are

(14) $$\begin{array}{c} u_{i}=\frac{\beta_{11}x_{i}+\beta_{12}y_{i}+\beta_{13}z_{i}+\beta_{14}}{\beta_{41}x_{i}+\beta_{42}y_{i}+\beta_{43}z_{i}+\beta_{44}},\\ v_{i}=\frac{\beta_{21}x_{i}+\beta_{22}y_{i}+\beta_{23}z_{i}+\beta_{24}}{\beta_{41}x_{i}+\beta_{42}y_{i}+\beta_{43}z_{i}+\beta_{44}},\\ w_{i}=\frac{\beta_{31}x_{i}+\beta_{32}y_{i}+\beta_{33}z_{i}+\beta_{34}}{\beta_{41}x_{i}+\beta_{42}y_{i}+\beta_{43}z_{i}+\beta_{44}},\\ \left[\begin{array}{c} u_{i}t\\ v_{i}t\\ w_{i}t\\ t \end{array}\right]=\left[\begin{array}{cccc} \beta_{11} & \beta_{12} & \beta_{13} & \beta_{14}\\ \beta_{21} & \beta_{22} & \beta_{23} & \beta_{24}\\ \beta_{31} & \beta_{32} & \beta_{33} & \beta_{34}\\ \beta_{41} & \beta_{42} & \beta_{43} & \beta_{44} \end{array}\right]\left[\begin{array}{c} x_{i}\\ y_{i}\\ z_{i}\\ 1 \end{array}\right]. \end{array}$$

Let

(15) $$\begin{array}{c} u_{i}^{\prime }=u_{i}t,\\ v_{i}^{\prime }=v_{i}t,\\ w_{i}^{\prime }=w_{i}t, \end{array}$$

then

(16) $$\left[\begin{array}{c} u_{i}^{\prime }\\ v_{i}^{\prime }\\ w_{i}^{\prime }\\ t \end{array}\right]=\left[\begin{array}{cccc} \beta_{11} & \beta_{12} & \beta_{13} & \beta_{14}\\ \beta_{21} & \beta_{22} & \beta_{23} & \beta_{24}\\ \beta_{31} & \beta_{32} & \beta_{33} & \beta_{34}\\ \beta_{41} & \beta_{42} & \beta_{43} & \beta_{44} \end{array}\right]\left[\begin{array}{c} x_{i}\\ y_{i}\\ z_{i}\\ 1 \end{array}\right],$$

and deriving the normal equations in the usual manner yields

(17) $$\left(I_{4}⊗\left[\begin{array}{cccc} \sum x^{2} & \sum xy & \sum xz & \sum x\\ \sum xy & \sum y^{2} & \sum yz & \sum y\\ \sum xz & \sum yz & \sum z^{2} & \sum z\\ \sum x & \sum y & \sum z & N \end{array}\right]\right)\left(\begin{array}{c} \beta_{11}\\ \beta_{12}\\ \beta_{13}\\ \beta_{14}\\ \beta_{21}\\ \beta_{22}\\ \beta_{23}\\ \beta_{24}\\ \beta_{31}\\ \beta_{32}\\ \beta_{33}\\ \beta_{34}\\ \beta_{41}\\ \beta_{42}\\ \beta_{43}\\ \beta_{44} \end{array}\right)=\left(\begin{array}{c} \sum ux\\ \sum uy\\ \sum uz\\ \sum u\\ \sum vx\\ \sum vy\\ \sum vz\\ \sum v\\ \sum wx\\ \sum wy\\ \sum wz\\ \sum w\\ \sum tx\\ \sum ty\\ \sum tz\\ \sum t\;\; \end{array}\right),$$

where **I**_4_ is a 4 × 4 identity matrix and ⊗ is the direct product of the two matrices.

As described in [4], this linearization results in parameter estimates that reduce the transformation to affine. The linearization is adequate to determine the transformed points, but not for the optimization to determine the transformation parameters or for measuring the degree of similarity between the two objects. Therefore, the transformation is left in terms of the original equations and nonlinear regression is used to obtain parameter estimates.

Nonlinear regression is an extension of linear regression where the expected responses are nonlinear functions of the parameters [13]. Finding least-squares estimates for linear models is straightforward as they have a closed-form solution. For nonlinear models, the least-squares estimates must be found using an iterative procedure. In this paper, the Gauss-Newton algorithm is used. This iterative procedure utilizes a Taylor series expansion to find the least-squares estimates [13].

For all transformations, parameter estimates can be found in the usual manner, ${\hat{\beta }}_{j}={\left(X_{j}^{T}X_{j}\right)}^{-1}X_{j}^{T}Y$, and subsequently used to solve for rotation, scale, and sheer parameters. The similarity of the two objects can be assessed using the tridimensional correlation coefficient, *R*_3*D*_, given by

(18) $$R_{3D}=\sqrt{1-\frac{\sum_{i}\left\{{\left(u_{i}-{\hat{u}}_{i}\right)}^{2}+{\left(v_{i}-{\hat{v}}_{i}\right)}^{2}+{\left(w_{i}-{\hat{w}}_{i}\right)}^{2}\right\}}{\sum_{i}\left\{{\left(u_{i}-\overset{̅}{u}\right)}^{2}+{\left(v_{i}-\overset{̅}{v}\right)}^{2}+{\left(w_{i}-\overset{̅}{w}\right)}^{2}\right\}}},$$

which is an extension to the bidimensional correlation coefficient [1].

## 3. Results and Discussion

**Figure 4:**
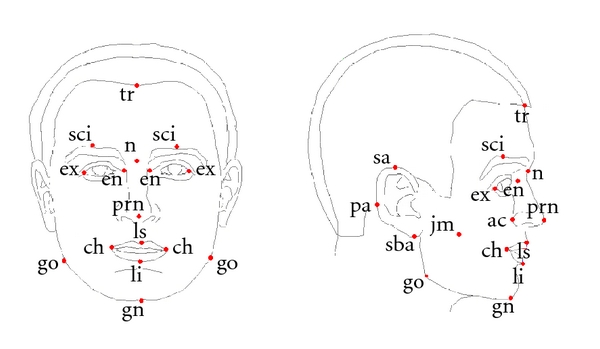
Landmarks used for evaluating tridimensional regression.

An experiment was conducted to evaluate the effectiveness of tridimensional regression and its improvement over bidimensional regression. Three-dimensional landmark data obtained from human faces were used for this purpose. The landmarks were obtained by placing reflective markers on the faces of subjects and tracking the coordinates as the subjects moved through a series of poses using automated software. The landmarks were adapted from [14]. They are shown in Figure 4 and described in Table 1.

The landmarks were obtained for three subjects at two different sittings and five poses per sitting. The objective was to compare *R* ^2^ values within a subject to the *R* ^2^ values between subjects using both tridimensional regression and bidimensional regression. One would expect the degree of similarity to be higher, thus a higher *R* ^2^ value, for two samples from the same person than for samples from two different people. All pairwise *R* ^2^ values were calculated for bidimensional and tridimensional regressions. Poses of the same individual within a sitting were not compared since the markers were not removed between poses and using these poses would result in inflated *R* ^2^ values. 

**Figure 5:**
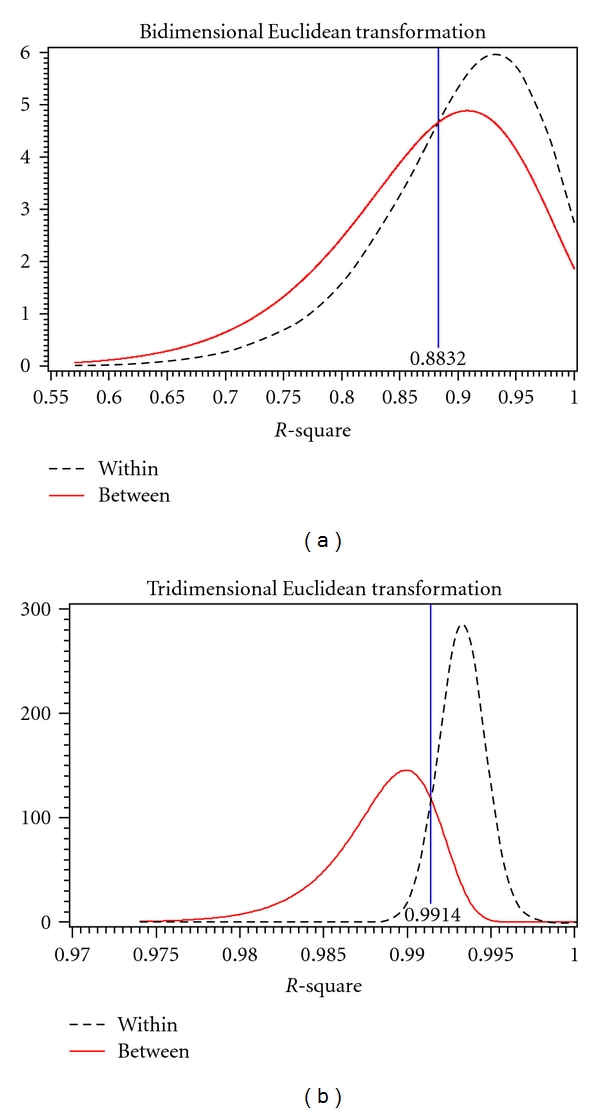
Within and between person *R* ^2^ for bidimensional (*l*) and tridimensional (*r*) Euclidean transformation.

**Figure 6:**
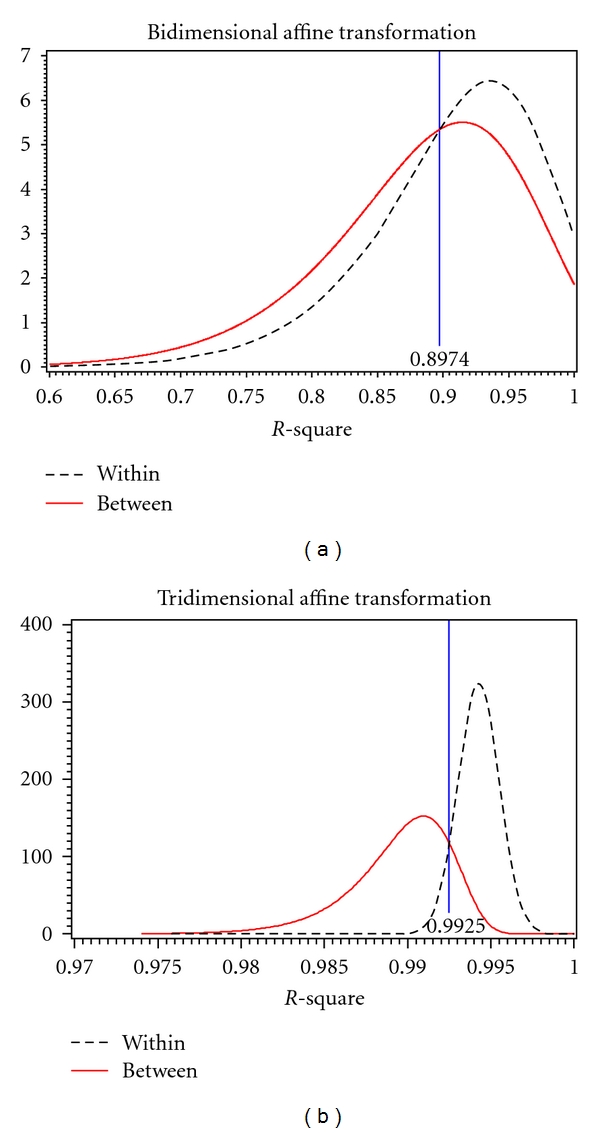
Within and between person *R* ^2^ for bidimensional (*l*) and tridimensional (*r*) affine transformation.

**Figure 7:**
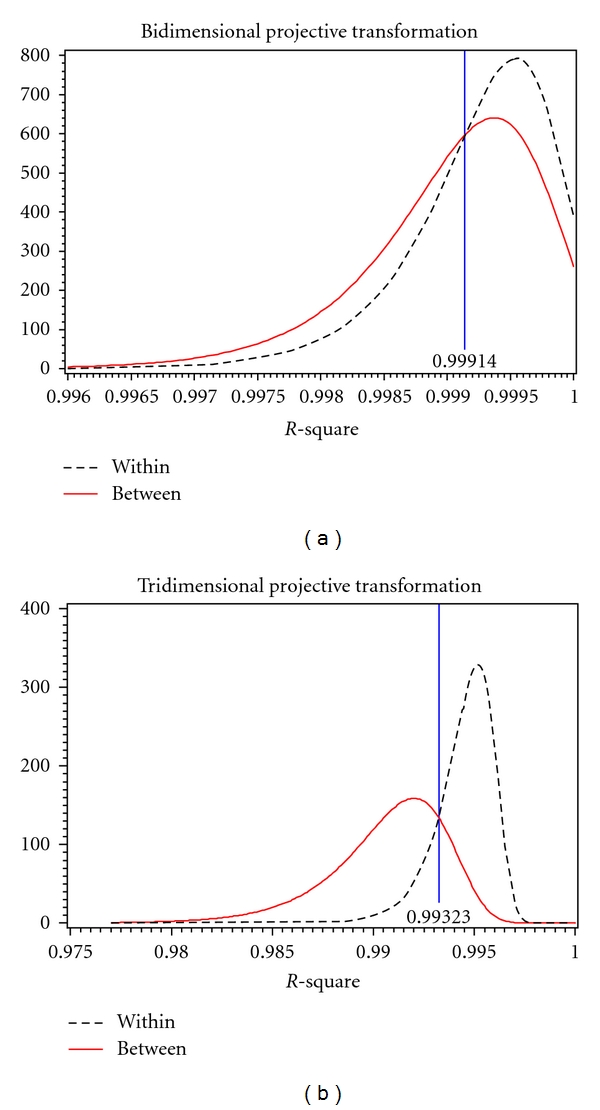
Within and between person *R* ^2^ for bidimensional (*l*) and tridimensional (*r*) projective transformation.

For each transformation, both in two and three dimensions, the distributions of *R* ^2^ values for within and between subjects were obtained by fitting a theoretical distribution over the histograms of observed values. Overlaying these theoretical distributions allowed for the estimation of a threshold value (*τ*) as a cutoff for determining if two images were from the same subject. *R* ^2^ values greater than *τ* lead to the decision that the two images are of the same subject (match) while *R* ^2^ values less than *τ* indicate that the images are of two different subjects (nonmatch). The threshold value was determined to be where the two distributions cross, as to simultaneously minimize the false-positive and false-negative error rates. A false positive is when images of two different subjects are incorrectly determined to be from the same subject (an *R* ^2^ value greater than *τ* for different subjects); a false-negative occurs when two images from the same subject are incorrectly determined to be from different subjects (an *R* ^2^ value less than *τ* for the same subject). In addition to calculating the observed error rates, the expected error rates were found by evaluating the cumulative distribution functions of the *R* ^2^ values at *τ*.  Table 2 summarizes the observed and expected error rates, and Figures 5, 6, and 7 show the within-subject (dotted line) and between subject (solid line) distributions for each transformation.

Table 2 shows that both the observed and expected error rates for tridimensional regression are much smaller than those for bidimensional regression using any of the three transformations. Bidimensional regression resulted in both error rates being very high, false-positives often over fifty percent. Tridimensional regression shows a substantial decrease in both false-positive and false-negative error rates which indicates that the three-dimensional method is better at correctly matching a subject to him or herself. 

In this application, the Euclidean and affine transformations were comparable to one another with the affine performing slightly better. The projective transformation had the largest observed false-positive rate. This result is not surprising as the flexibility of the projective transformation allows it to map objects into many other shapes. This flexibility results in the ability to match even two very dissimilar objects quite well with certain transformation parameters. Consequently, the *R* ^2^ values are very high for all matches. This shifts the between-person distribution closer to the within person-distribution which results in a larger false-positive error rate. 

Additionally, a sixth pose was taken on each of the subjects in each setting. This pose was not used to build the within- and between-subject distributions, or to determine the threshold. These six sets of points (two for each subject) were compared to all other poses not taken in the same setting of the same subject (30 comparisons per pose, 6 possible correct matches). The highest *R* ^2^ for all six was a correct match. In addition, a minimum of the top 4 matches were correct matches, illustrating that tridimensional regression can be very good at identifying correct matches and discriminating between different objects.

## 4. Conclusion

Bidimensional regression [1] is a useful tool for comparing two geometric configurations that are each represented by a set of coordinate pairs. The scale, rotation, and translation relating the two configurations can be estimated by first estimating the parameters of the transformation model. As an application of the technique, [2, 3] used bidimensional regression analysis for relating faces in landmark-based face recognition. 

In this paper, the bidimensional technique has been extended to three dimensions. Such an extension may prove useful in the analysis of three-dimensional landmark data. The underlying foundations for tridimensional regression have been developed with different transformations: Euclidean, affine, and projective. Its use is demonstrated through an application to compare human faces using three-dimensional landmarks. Results show that tridimensional regression improves the ability to correctly match objects that are represented by landmark data. Both the Euclidean and affine transformations work well to reduce the error rates. The projective transformation also shows improved error rates, but its flexibility may make it too general for some practical applications. Choice of transformation should be given careful consideration given the goals of the application. While there is improvement over Bidimensional regression, the observed and expected error rates are likely higher in this experiment due to the small number of subjects involved and comparing several poses of the same subject. A larger-scale study is needed to better estimate the expected error rates.

This work can be extended in several different directions. The focus here was in developing the theory of tridimensional regression and conducting an initial investigation for shape matching with a feasibility experiment. An investigation with a larger amount of three-dimensional landmark data is needed to more fully understand its effectiveness. In addition to a larger-scale study, it is also of interest to develop weighted tridimensional regression techniques which would allow some landmarks to be weighted more or less heavily than others. Weighting landmarks allows for less weight to be placed on landmarks that are highly variable. Some landmarks could be more variable because they are less reliably extracted or simply due to more natural variability. Weighting has been shown to improve the matching ability in bidimensional regression [15], specifically in a face matching application [16], and is expected to improve the matching ability and precision in mapping for tridimensional regression as well.

## Figures and Tables

**Table 1:** Description of landmarks used for evaluation (adapted from [14]).

tr	The point on the hairline in the midline of the forehead.
go	The most lateral point on the mandibular angle close to the bony gonion.
gn	The lowest median landmark on the lower border of the mandible.
en	The point at the inner commissure of the eye fissure.
ex	The point at the outer commissure of the eye fissure.
sci	The highest point on the upper boarder in the midportion of each eyebrow.
*n*	The midpoint of both the nasal root and the nasofrontal structure.
prn	The most protrudent point of the apex nasi.
ac	The most lateral point in the curved baseline of each ala.
ls	The midpoint of the upper vermillion line.
li	The midpoint of the lower vermillion line.
ch	The point located at each labial commissure.
sa	The highest point of the free margin of the auricle.
sba	The lowest point of the free margin of the ear lobe.
pa	The most posterior point on the free margin of the ear.
jm	The most protrudent point of the muscle when the jaw is clenched.

**Table 2:** Error rates for each transformation.

		Bidimensional regression		Tridimensional regression	
		False positive	False negative	False positive	False negative
Euclidean	Observed	59.7%	36.0%	19.3%	12.0%
	Expected	51.9%	33.6%	16.7%	9.0%
Affine	Observed	57.5%	38.7%	17.0%	3.3%
	Expected	49.0%	37.2%	14.9%	7.5%
Projective	Observed	56.8%	35.3%	23.5%	7.3%
	Expected	51.2%	34.5%	18.4%	16.2%
